# Unveiling the Shape of *N*-Acetylgalactosamine: A Cancer-Associated Sugar Derivative

**DOI:** 10.1021/acs.jpca.2c04595

**Published:** 2022-09-13

**Authors:** R. Aguado, M. Sanz-Novo, S. Mata, I. León, J. L. Alonso

**Affiliations:** † Grupo de Espectroscopía Molecular (GEM), Edificio Quifima, Área de Química-Física, Laboratorios de Espectroscopía y Bioespectroscopía, Parque Científico UVa, Unidad Asociada CSIC, Universidad de Valladolid, Valladolid 47011, Spain

## Abstract

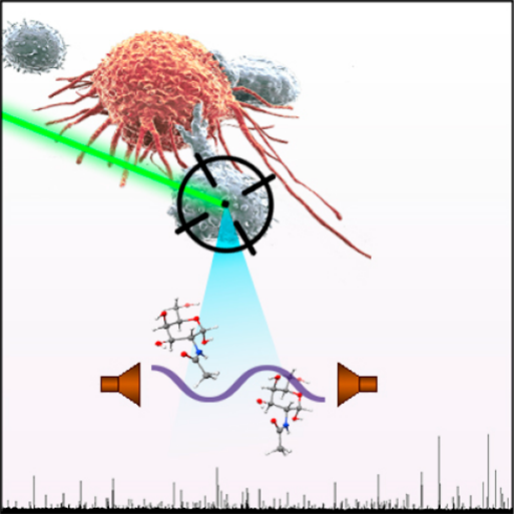

In the present work, we report the first rotational study of *N*-acetylgalactosamine, a cancer-associated sugar derivative, by means of high-resolution rotational spectroscopy. Two different conformers have been conclusively characterized using broadband Fourier transform microwave spectroscopy coupled with a laser ablation vaporization system. Additionally, we performed a comprehensive analysis of the intramolecular interactions that govern these structures, which allowed us to both characterize the existence of intramolecular hydrogen bond networks that drive the intrinsic conformation panorama of *N*-acetylgalactosamine and further rationalize the biological role of this aminosugar derivative as part of the Tn antigen.

## Introduction

**Figure 1:**
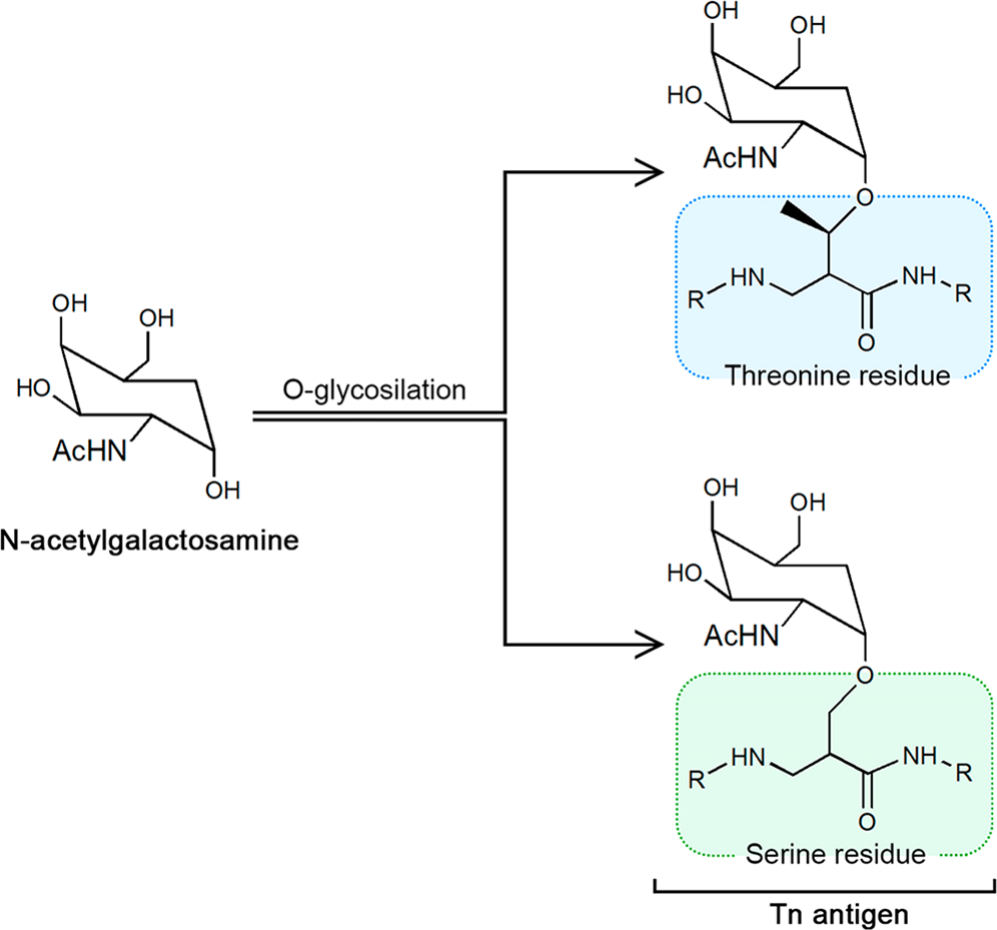
Schematic representation of GalNAc and the Tn antigen (with serine and threonine linked to the sugar, respectively). The α-form, which exhibits the anomeric hydroxyl group in an axial disposition, is shown for all structures because it is the bioactive form.

*N*-Acetylgalactosamine (GalNAc, shown in Figure 1) is an amino sugar derivative of galactose that plays an essential role in different biological processes inside the human body. One of the most relevant roles of *N*-acetylgalactosamine lies in the formation of the Tn antigen,^1,2^ a molecular structure related to metastatic processes. This antigen results from the binding between a GalNAc molecule and a serine or threonine residue in the extracellular domain of a mucin. As illustrated in Figure 1, the binding happens through an *O*-glycosidic bond and constitutes the first step in the glycosylation process.^3^

A recent study has shown that the overexpression and exposition of GalNAc molecules on the cell membrane is clinically associated with cancer metastasis^1^ due to a failure in the protein glycosylation process that leads to the exposition of Tn antigen, which has been associated with an increased potential for invasion and metastasis of cancer cells.^2^ Researchers have carried out a significant number of clinical studies to shed some light on the relationship between the exposition of the Tn antigen and the metastatic processes, concluding that the overexpression of this molecule is undoubtedly associated with poor patient prognosis and the development of metastasis in a wide range of cancers.^2^ The Tn antigen favors tumor growth and the development of metastatic processes, since cancer cells can use the Tn antigen to invade other tissues through noncovalent interactions with lectins of the target tissue.^1,2,4^ Due to its crucial role in metastasis, the Tn antigen is known as the most specific human-cancer-associated structure.^5,6^

Despite its fundamental importance in understanding different cancer-associated processes, the study of *O*-GalNAc glycosylation stands as a challenging task for the scientific community because of a lack of specific tools for biological assays. All structural information regarding GalNAc is reduced to condensed-phase studies through infrared or X-ray spectroscopic techniques.^7−9^ However, the structural data extracted from these investigations are somewhat limited, since they can be perturbed by disturbing agents such as the solvent or other molecules in the crystal. Therefore, as a first step to better understand the Ser(O)- or Thr(O)-linked glycosylation with *N*-acetylgalactosamine, it is necessary to first unravel the naked structure of GalNAc under gas-phase isolation conditions, which will also allow us to evaluate the intramolecular interactions that govern its intrinsic conformational properties. Rotational spectroscopy stands as an unrivaled technique to achieve this challenging endeavor. On the one hand, Fourier transform (FT) microwave spectroscopy allows a detailed description of the three-dimensional structure by obtaining the rotational parameters directly related to the molecular geometry. On the other hand, the use of supersonic jets allows the target molecule to be probed in an isolated environment free from collisions or interactions with any surrounding species.

Nevertheless, GalNAc is a solid with a high melting point (mp 172–173 °C) that cannot be vaporized by the standard heating methods due to its high inherent thermolability. Our group developed laser ablation techniques to overcome this limitation while transferring solid biomolecules to the gas phase.^10,11^ This methodology, coupled with state-of-the-art FTMW spectrometers, has allowed us to characterize a wide variety of biomolecules, highlighting those intimately related to GalNAc, such as galactose, glucosamine, and glucose.^12−14^ The rotational study of GalNAc has remained unfeasible until now due to the intrinsic chemical instability of the molecule. Recent improvements in the sample preparation procedure and careful control of the fragmentation processes that occur because of laser ablation^15,16^ have enabled its first rotational characterization.

We present the first high-resolution spectroscopic study of this relevant amino sugar derivative. The latest generation of laser ablation chirped-pulse Fourier transform microwave (LA-CP-FTMW) spectrometers^17^ has been used to probe the GalNAc in an isolated environment. With this approach, we seek to characterize not only the molecular structure but also the intramolecular forces that govern the most stable structures of GalNAc. This relevant information shall ultimately lead to a better interpretation of the interaction between lectins and the Tn antigen.

## Experimental Methodology

We used a commercial sample of GalNAc (Glentham Live Sciences, >99%) without further purification to form solid rods by pressing a mixture of the compound’s fine powder and a small amount of a commercial copolymeric binder. To record the rotational broadband spectrum, we used a LA-CP-FTMW spectrometer designed to maximize performance in the study of large biomolecules.^18^ A GalNAc rod was placed in the ablation nozzle and vaporized using the fourth harmonic of a picosecond Nd:YAG laser.^14,19^ The ablation products were supersonically expanded using a neon flow (backing pressure of 10 bar) and then probed by CP-FTMW spectroscopy in the 6–14 GHz region. We employed chirped pulses of 4 μs, directly generated by a 24 GS·s^–1^ arbitrary waveform generator, that were amplified to about 300 W peak power using a traveling wave tube (TWT) amplifier. Two dual-ridge horns broadcasted the excitation pulse and received the broadband molecular emission. At a repetition rate of 2 Hz, up to 137 000 free induction decays were averaged and digitized using a 50 GS·s^–1^ digital oscilloscope. Finally, the time-domain spectrum was Fourier-transformed to obtain the broadband spectrum in the frequency domain. The experimental uncertainty of the unblended symmetric lines was estimated to be about 20 kHz, and the frequency resolution was typically of ∼100 kHz.

## Results and Discussion

### Computational Modeling

**Figure 2:**
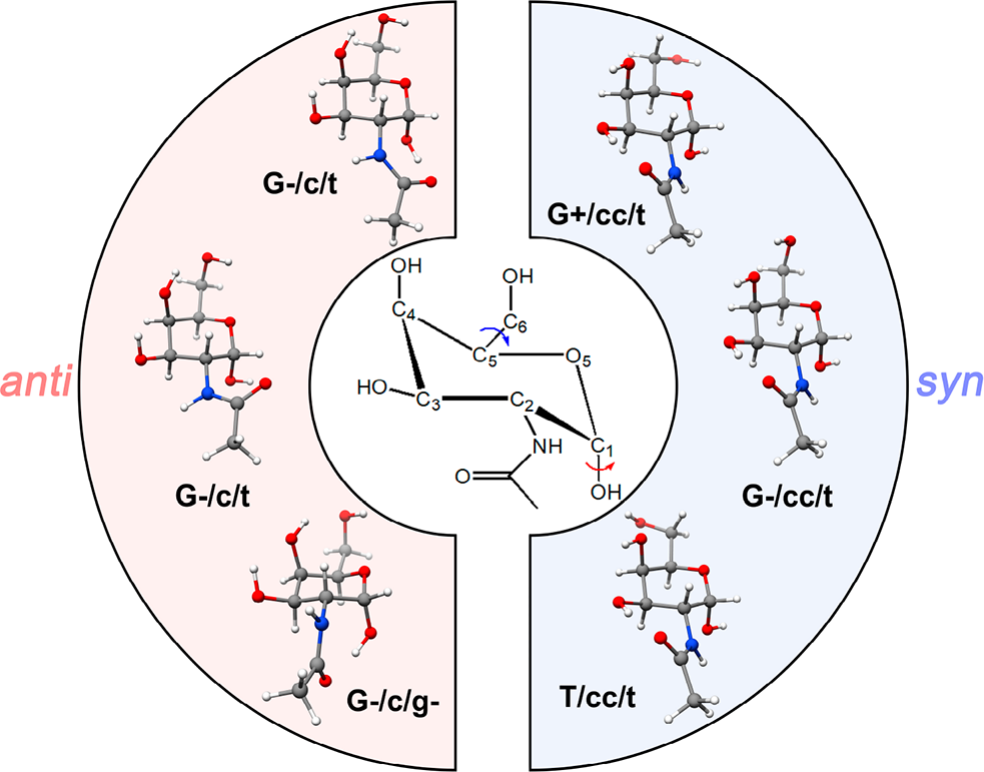
Six low-lying structures for GalNAc at the B2PLYP-D3BJ/6-311++G(d,p) level of theory. The conformers are grouped in the two *anti/syn* families.

To facilitate the analysis of the rotational spectrum, we first explored the conformational panorama of GalNAc. This biomolecule presents four hydroxyl groups and one *N*-acetyl moiety (see Figure 2), which can lead to a vast conformational space. Therefore, we performed a thorough conformational search using a combination of fast molecular mechanics methods. The static Merk molecular force field (MMFFs)^20^ was used in combination with two search algorithms—the “Large scales Low Mode” and a Monte Carlo-based search—as implemented in Macromodel.^21^ This conformational search led to a total of 55 different structures within an energy window of 30 kJ·mol^–1^, which were optimized first at the B3LYP-D3BJ/6-311++G(d,p) level of theory.^22−24^ Each optimized structure was confirmed to be a local minimum on the potential energy surface by checking that its Hessian matrix did not have any imaginary eigenvalues. Then, structures below 700 cm^–1^—those conformers likely to be populated in the supersonic expansion— were reoptimized using a double-hybrid B2PLYP-D3BJ functional,^25^ which includes a Grimme dispersion and Becke–Johnson damping^26^ in combination with Pople’s triple-ζ basis set.^27^ All geometry optimizations were done using the Gaussian 16 program package.^28^ Other higher-level calculations were carried out as a computational benchmark for this type of biomolecular system, and their results are summarized in the Supporting Information.

The modeled structures can be sorted into two families (see Figure 2): the *syn* family contains those conformers in which the orientation of the *N*-acetyl moiety allows the carbonyl group to interact with the hydroxyl group in position 3 through an intramolecular hydrogen bond, while the *anti* family presents the *N*-acetyl moiety in an opposite disposition, with the carbonyl group interacting with the anomeric hydroxyl group and the N–H group establishing an electrostatic interaction with the hydroxyl group in position 3. Interestingly, theoretical calculations predict three low-lying-in-energy conformers for each family, with those of the *syn* family being the most stable ones. The predicted spectroscopic parameters for all of them are summarized in the first section of Table 1.

**Table 1:** Theoretically Predicted and Experimental Rotational Parameters for the Observed Rotamers of GalNAc

	*syn*/G+/cc/t^a^	*syn*/G-/cc/t	*syn*/T/cc/t	*anti*/G+/c/g-	*anti*/G+/cc/t	*anti*/G-/c/t	rotamer I	rotamer II
*A*^b^	1205.9	1188.8	1216.1	1075.4	1230.7	1169.0	1211.0116(31)^j^	1220.9325(41)
*B*	364.1	374.9	366.6	379.9	391.6	385.8	363.8535(36)	365.3756(25)
*C*	301.7	315.9	304.9	335.5	336.2	319.7	303.0151(15)	305.8039(29)
μ_a_^c^	2.3	3.2	3.1	1.0	0.8	0.1	observed^k^	
μ_b_	4.6	4.4	4.6	0.7	1.1	1.3	observed	observed
μ_c_	0.7	2.3	0.5	2.7	2.3	0.0		
χ_aa_^d^	2.361	2.417	2.279	2.513	2.363	2.402		
χ_bb_	0.583	0.846	0.574	–0.183	–2.290	1.639		
χ_cc_	–2.944	–3.263	–2.853	–2.330	–0.073	–4.040		
Δ*E*^e^	0	173	221	542	409	450		
Δ*E*_ZPE_^f^	0	234	317	507	594	637		
Δ*G*^g^	0	317	311	327	736	751		
*N*^h^							40	15
σ_RMS_^i^							43	51

a The most stable conformers resulting from geometric optimization computed at the B2PLYP-D3BJ/6-311++G(d,p) level of theory.

b *A*, *B*, and *C* are the rotational constants (MHz).

c μ_a_, μ_b_, and μ_c_ are the absolute values of the dipole moment (debyes).

d χ_aa_, χ_bb_, and χ_cc_ are the diagonal elements of the ^14^N nuclear quadrupole coupling tensor (MHz);

e Electronic energies (cm^–1^).

f Electronic energies with a zero-point correction at the same level of calculation (cm^–1^).

g Gibbs free energies at 298 K and the same level of calculation (cm^–1^).

h The number of measured transitions.

i Root-mean-square deviation of the fit (kHz).

j The numbers in parentheses are the 1σ uncertainties in units of the last decimal digit.

k Experimental observation of a given type of rotational transition.

We used a notation based on four different symbols for easier reading: (a) the prefix *anti* or *syn* indicates the family of each conformer, (b) A capital letter, namely, G+, G-, or T, is used to describe the *gauche*- or *trans*-configuration of the ∠O_6_–C_6_–C_5_–O_5_ dihedral angle, (c) lowercase letters, namely, *c* or *cc*, are used describe the orientations of the hydroxyl groups in positions 3 and 4, which can be clockwise (*c*) or counterclockwise (*cc*), and (d) a lowercase letter, namely, *g+*, *g-*, or *t* is used to describe the *gauche*- or *trans*-configuration of the ∠H_1_–O_1_–C_1_–C_2_ dihedral angle.

### Broadband LA-CP-FTMW Spectrum

**Figure 3:**
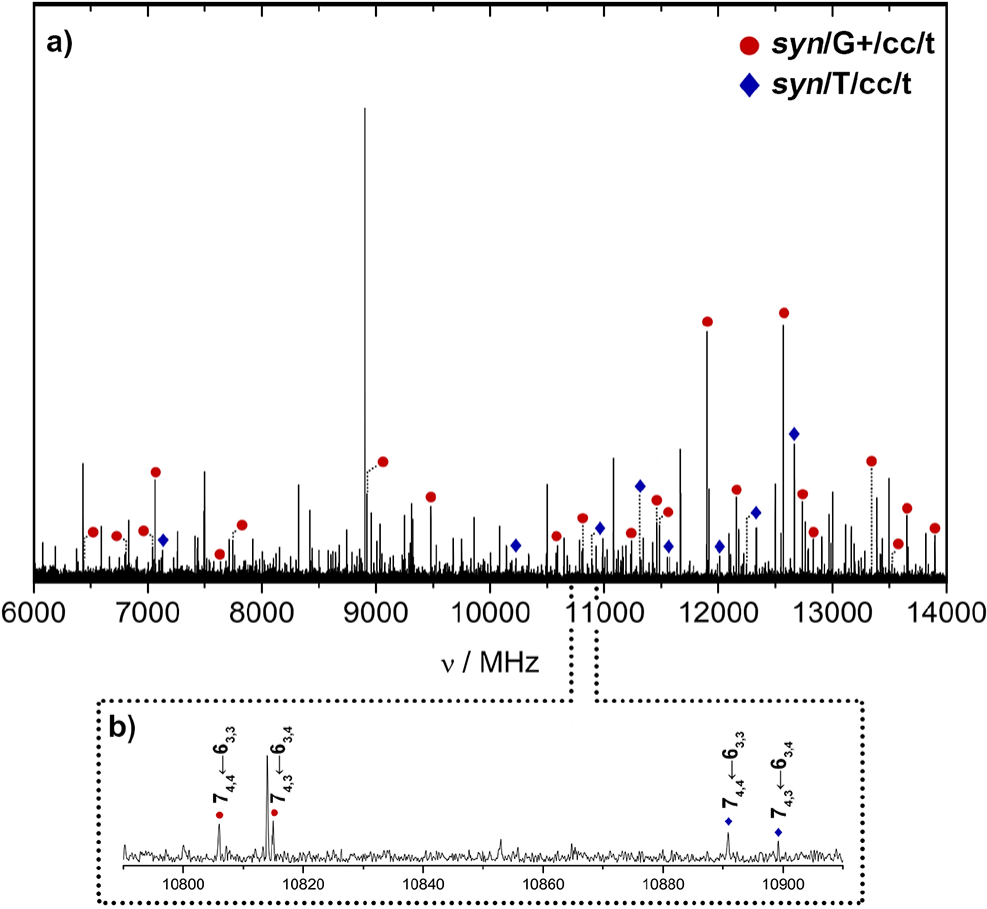
(a) LA-CP-FTMW spectrum of GalNAc obtained in the 6.0–14.0 GHz frequency region. The two identified rotamers are marked in red and blue, respectively. (b) Close-up view showing an example of the pairs of *b*-type transitions that helped the identification of GalNAc during the analysis.

The broadband jet-cooled rotational spectrum of GalNAc in the 6–14 GHz frequency region is shown in Figure 3. The spectrum is dense with a plethora of low-intensity lines, many of which are broadened by ^14^N nuclear quadrupole coupling effects arising from the ^14^N nucleus of the *N*-acetyl group. This nucleus presents a nonzero nuclear quadrupole moment that interacts with the electric field gradient created by the rest of the molecule at the nuclei. The interaction splits the rotational energy levels, giving rise to a very complex hyperfine structure. Thus, the intensity of each rotation transition is distributed among the quadrupole components, making the detection and analysis of these transitions difficult. Additionally, the spectral resolution in the CP-FTMW experiments was insufficient to resolve these quadrupole hyperfine structures completely, and only frequency centers were considered in the analysis.

All six low-energy conformers of GalNAc in Table 1 were predicted to be near prolate asymmetric rotors with sizable values of the dipole moment components. We first examined the spectrum by looking for a series of μ_a_-type *R*-branch transitions spaced by approximately *B + C*. We identified several progressions corresponding to higher values of *K* (e.g.,  = 4 transitions shown in Figure 3b). This first set of transitions was fitted to a rigid rotor Hamiltonian,^29^ providing an initial set of rotational constants; these values were used to make more accurate predictions. We followed an iterative fitting procedure and measured up to 40 μ_b_-type and μ_a_-type *R*-branch transitions, which are listed in Table S1 of the Supporting Information. The rotational parameters for the first rotamer are collected in the second section of Table 1. Afterward, we removed the rotational transitions belonging to this species and analyzed the remaining lines looking for a second rotamer. We then assigned 15 weak μ_b_-type *R*-branch transitions to a second rotamer II, which was also fitted to a rigid rotor Hamiltonian; this procedure provided the second set of rotational constants listed in Table 1. Note that the standard deviation of the fit is somewhat larger than expected due to the effect of the nuclear quadrupole coupling and the inclusion of partially resolved lines (intensity-weighted mean of the hyperfine line cluster) in the fit.

Regarding the conformational identification of the observed rotamers, although this process is not always trivial, we can achieve a conclusive identification by matching the experimental rotational constants with those DFT-predicted for the lowest-lying structures, as shown in Figure 3. Therefore, we easily ascribed rotamer I to the *syn*/G+/cc/t conformer and rotamer II to the *syn*/T/cc/t conformer. This assignment was further corroborated using the trend in the values of the rotational constants while going from rotamer I to rotamer II, which is only coherent with the predicted changes while traveling from *syn*/G+/cc/t to *syn*/T/cc/t GalNAc. Scaling factors ranging from 0.996 to 1.003 bring the predicted B2PLYP-D3BJ rotational constants values in agreement with the experimental ones, supporting the reliability of the conformational assignment.

According to the predicted energy difference between the conformers (see Table 1), the observation of the *syn*/T/cc/t conformer suggests that the *syn*/G-/cc/t conformer could also be populated enough to be detected. Consequently, we eliminated the lines assigned to *syn*/G+/cc/t and *syn*/T/cc/t GalNAc and performed thorough searches around the predicted transitions for the *syn*/G-/cc/t conformer using the aforementioned scaling factors. Unfortunately, no spectral signatures attributable to other rotameric species of GalNAc were detected. Nevertheless, we note that transferring the GalNAc molecules from the solid into the gas phase turned out to be a challenging task. We must carefully adjust the experimental parameters (laser fluence, laser wavelength, backing pressure, etc.) to minimize the fragmentation processes. Despite all our efforts, much fragmentation still took place, which minimized the generation of neutral GalNAc molecules (see Figure 3a). Consequently, the transitions of the *syn*/T/cc/t conformer, which is higher in energy, are extremely weak and barely arise from the background noise level (see the transitions marked with blue color in Figure 3b). Therefore, transitions corresponding to the *syn*/G-/cc/t conformer could be of slightly lower intensity than those ascribed to the *syn*/T/cc/t conformer and would be therefore present below the 3σ level, precluding its conclusive detection. These weak rotational lines may also appear even more weakened by the effect of the ^14^N nuclear quadrupole coupling, as mentioned previously, which further complicates the analysis. Finally, both *syn*/G-/cc/t and *syn*/T/cc/t conformers are predicted to be almost isoenergetic; therefore, a slight variation in the calculation errors could alter their relative stabilities. In fact, the use of different methodologies (see Table S1 of the Supporting Information) provides different energetic values. Altogether, this helps us rationalize the nondetection of the *syn*/G-/cc/t conformer in the experiment. Additionally, a comparison between the gas-phase and solid phase structures^8,29^ highlights a slight tilt of the *N*-acetyl moiety in the opposite direction to that observed in our experiment for the isolated structures. This discrepancy should result from the isolation conditions achieved with the supersonic expansion, where the conformational panorama is ruled only by intramolecular forces; this will be comprehensively investigated in the next section.

### The Role of the Intramolecular Interactions

**Figure 4:**
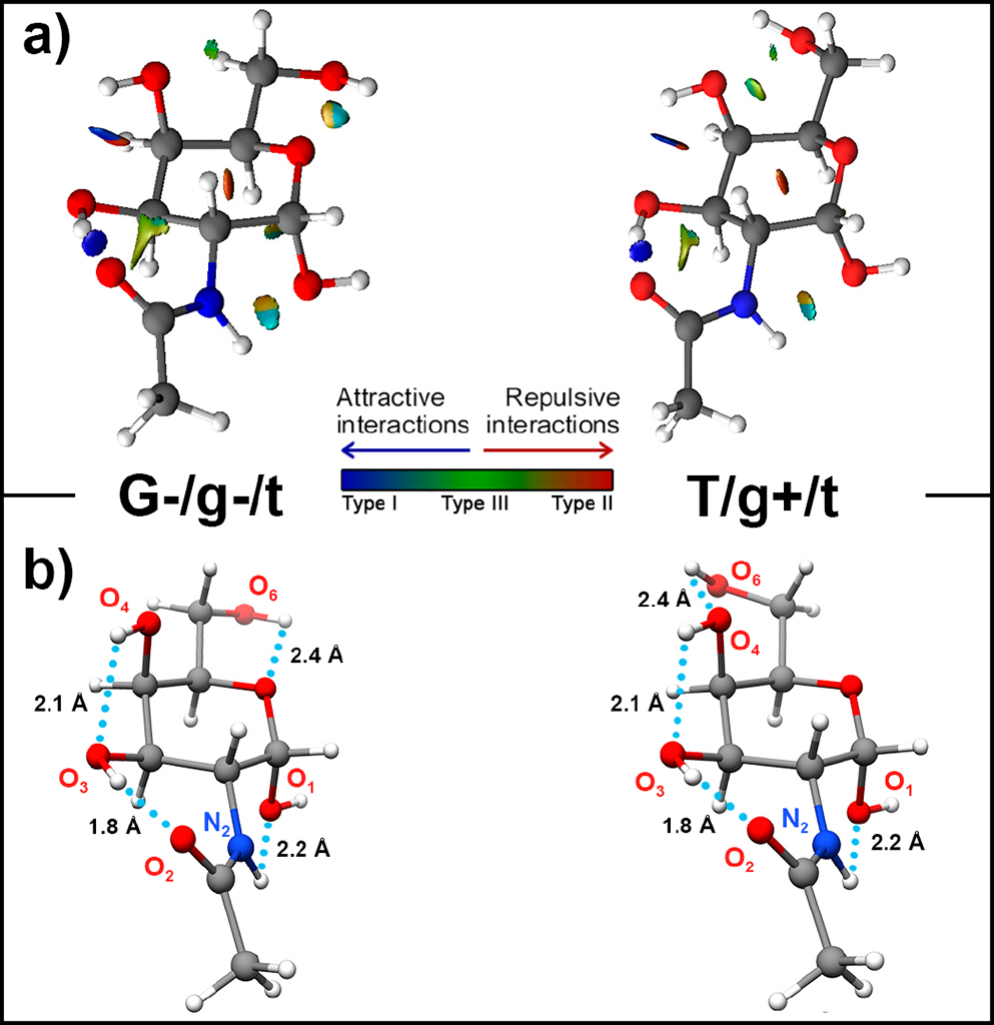
(a) NCI representations of the *syn*/G+/cc/t and *syn*/T/cc/t conformers of GalNAc, which were obtained using VMD software.^33^ Different NCI-type isosufaces were found during the analysis: NCI type I isosurfaces are colored in blue and correspond to strong stabilizing interactions (such as hydrogen bonds), NCI type II isosurfaces are represented in red and account for strong destabilizing interactions, (i.e., steric crowding), and NCI type III interactions are represented in green and gather the delocalized weak van der Waals interactions. The isovalue is 0.3 au. (b) Modeled structure of the detected conformers of GalNAc. Intramolecular hydrogen bonds that stabilize the structures are highlighted as dot lines.

Once we had completed the analysis of the conformational panorama of the GalNAc molecule, we performed a comprehensive study of the intramolecular interactions stabilizing the observed structures to probe the nature of the intramolecular bonding. We carried out a noncovalent interactions (NCI) analysis^30^ based on the B2PLYP-D3BJ/6-311++G(d,p) structures using the NCIPLOT4 software.^31^ This analysis allows us to visualize weak noncovalent interactions from the topological analysis of the electron density (*r*) and its reduced gradient.^32^ The representation of the different intramolecular noncovalent interactions for the detected conformers is shown in Figure 4.

The two characterized structures belong to the *syn* family of conformers and present the *N*-acetyl moiety tilted toward the OH_(3)_ hydroxyl group. This disposition forces the OH_(3)_ group to interact with the *N*-acetyl moiety through a strong O_(3)_—H_(3)_···O=C hydrogen bond interaction (see Figure 4). Thus, the relative disposition of the hydroxyl group is locked by such a hydrogen bond, forcing an O_(3)_–H_(3)_···O_(4)_–H_(4)_ interaction to take place. In addition, the tilt of the *N*-acetyl moiety also leads to an electrostatic interaction between the amino group and the anomeric hydroxyl group. The above-mentioned interactions are common for both detected conformers of GalNAc; the only difference between these two structures is the disposition of the hydroxymethyl group, which acts as a hydrogen bond donor in both conformers. For the *syn*/G-/cc/t conformer, this group establishes an O_(6)_–H_(6)_···O interaction with the oxygen from the heterocycle, while for the *syn*/T/cc/t conformer the interaction of the hydroxymethyl group is established with the OH_(4)_ hydroxyl group.

As it has been described, all the functional groups (OH and NH) of the molecule act as a donor in at least one hydrogen bond except for the anomeric hydroxyl group, which acts as a hydrogen bond acceptor. As a result, cooperative hydrogen bonds are established between vicinal hydroxyl groups to stabilize the structures, just as it has been detected for galactose^5^ and other sugars and sugar-derivatives.^6,8^

Attending to the strength of the interactions, the O_(3)_—H_(3)_···O=C hydrogen bond is the strongest, while the N–H···O_(1)_–H_(1)_ interaction is the weakest. This is an important aspect that can explain its biological role. As mentioned above, it is known that the Tn antigen has a molecular structure composed of a GalNAc molecule linked to a serine or threonine residue of a protein through an *O*-glycosidic bond. Interestingly, we show how this OH group at the anomeric position—where the formation of that bond will occur—does not act as a proton donor and that the N–H···O_(1)_–H_(1)_ interaction is very weak. Therefore, any chemical attack on this hydroxyl group requires a lower energetic cost than “breaking” a strong hydrogen bond. In other words, this bond’s formation reaction will be carried out through the position that requires lower energy consumption.

A final remark supporting this discussion is the formation process of antifreeze glycosylated proteins (AFGP),^34,35^ a class of glycoproteins that act as biological antifreeze agents in certain species of fishes, insects, bacteria, fungi, and plants.^36^ These proteins are typically composed of repeating Thr–Ala–Ala units that appear glycosylated through the threonine residue with the disaccharide β-d-galactosyl-(1 → 3)-α-d-*N*-acetylgalactosamine. This process is similar to the Tn antigen formation process, since the linkage of the disaccharide to the protein is also realized through the anomeric hydroxyl group.

## Conclusions

In the present work, we provide a thorough structural investigation of GalNAc, a relevant cancer-associated sugar derivative. Hence, its unbiased structure has been revealed for the first time in the isolation conditions granted by a supersonic jet using LA-CP-FTMW spectroscopy. The analysis of its broadband jet-cooled rotational spectrum has allowed us to characterize unequivocally two different structures for this molecule. Thus, the comprehensive structural information presented in this work could lead to a better understanding of the role of GalNAc in cancer biochemistry.

An exhaustive analysis of the intramolecular interactions has been carried out for the detected structures, revealing in both cases that an intramolecular hydrogen bond network is formed as a result of different O–H···O–H and O—H···O=C contacts. This type of interaction is analogous to those detected for other sugars and derivatives and plays a crucial role in stabilizing the detected conformers.

Finally, it is worth mentioning that all the obtained structural data are coherent with the biological role of GalNAc biomolecule, since for both detected rotamers the anomeric hydroxyl group is involved in the weakest intramolecular interaction. This fact further helps us to rationalize that the *O*-glycosilation process is carried out at this position.

## Supporting Information Available

Theoretical spectroscopic parameters and measured frequencies of the G+/g-/t and T/g+/t conformers of GalNAc (PDF)
