# Supplementary material for: Unveiling the Shape of N-Acetylgalactosamine: A Cancer-Associated Sugar Derivative
Source: J Phys Chem A. 2022 Sep 13;126(42):7621–6. doi: 10.1021/acs.jpca.2c04595 (PMC9620139; doi:10.1021/acs.jpca.2c04595)
Supplement: Supplementary file 1 — jp2c04595_si_001.pdf [file jp2c04595_si_001.pdf]

Supporting information for:

Unveiling The Shape Of N-Acetylgalactosamine: A Cancer-Associated Sugar Derivative

R. Aguado[†], M. Sanz-Novo[†], S. Mata[†], I. León[†] and J. L. Alonso[†,*]

†: Grupo de Espectroscopía Molecular (GEM), Edificio Quifima, Área de Química-Física, Laboratorios de Espectroscopía y Bioespectroscopía, Parque Científico UVa, Unidad Asociada CSIC, Universidad de Valladolid, 47011, Valladolid (Spain)

E-mail: jlalonso@qf.uva.es

Table S1. Theoretical spectroscopic parameters for the three low-lying conformers of GalNAc at different levels of theory.

***syn*/G+/cc/t** [a]	*A* [a]	*B* [a]	*C* [a]	μ_a	μ_b	μ_c	ΔE	ΔE_{ZPE}	ΔG
B3LYP-D3BJ 6-311++G(d,p)	1200.6	361.2	299.9	2.2	4.6	0.6	0	0	0
B2PLYP-D3BJ 6-311++G(d,p)	1205.9	364.1	301.7	2.3	4.5	0.7	0	0	0
MP2 6-311++G(d,p)	1212.8	364.9	303.7	2.3	4.5	0.7	0	0	0
BHandHLYP 6-311++G(d,p)	1218.1	367.4	304.0	2.3	4.6	0.7	212	247	424
***syn*/G-/cc/t**	*A* [a]	*B* [a]	*C* [a]	μ_a	μ_b	μ_c	ΔE	ΔE_{ZPE}	ΔG
B3LYP-D3BJ 6-311++G(d,p)	1179.9	372.4	314.2	3.1	4.4	2.2	98	140	244
B2PLYP 6-311++G(d,p)	1188.8	374.9	315.9	3.2	4.4	2.3	173	234	317
MP2 6-311++G(d,p)	1191.9	376.3	318.4	3.1	4.1	2.4	190	245	320
BHandHLYP 6-311++G(d,p)	1204.2	377.7	317.8	3.2	4.4	0.5	380	495	759
***syn*/T/cc/t**	*A* [a]	*B* [a]	*C* [a]	μ_a	μ_b	μ_c	ΔE	ΔE_{ZPE}	ΔG
B3LYP-D3BJ 6-311++G(d,p)	1209.5	363.6	302.9	3.0	4.5	0.5	163	244	237
B2PLYP 6-311++G(d,p)	1216.1	366.6	304.9	3.1	4.6	0.7	221	317	311
MP2 6-311++G(d,p)	1220.6	368.2	307.3	3.0	4.5	0.5	166	271	286
BHandHLYP 6-311++G(d,p)	1233.9	368.5	306.5	3.0	4.6	0.5	259	369	518

Table S2. Measured frequencies (values in MHz) for the rotational transitions of the *syn*/G+/cc/t conformer of GalNAc

J'	K'_{-1}	K'_{+1}	*J''*	K''_{-1}	K''_{+1}	ν_{obs}	$\nu_{obs} - \nu_{calc}$
10	1	10	9	0	9	6439.857	-0.017
11	0	11	10	1	10	6804.337	0.020
11	1	11	10	0	10	7003.403	0.019
4	3	2	3	2	1	7047.516	-0.048
12	0	12	11	1	11	7441.416	0.013
11	1	10	10	1	9	7441.860	-0.040
12	1	12	11	0	11	7578.046	-0.019
5	3	2	4	2	3	7745.381	0.051
10	2	9	9	1	8	8003.063	0.202
13	0	13	12	1	12	8069.241	0.001
6	3	4	5	2	3	8323.379	-0.002
6	3	3	5	2	4	8439.581	0.036
14	1	13	13	2	12	8659.440	-0.072
4	4	1	3	3	0	8810.884	-0.060
12	2	11	11	1	10	8851.048	-0.041
7	3	5	6	2	4	8922.443	-0.010
13	2	12	12	1	11	9286.553	-0.007
15	0	15	14	1	14	9306.545	0.040
14	1	13	13	1	12	9273.657	0.124
5	4	2	4	3	1	9478.231	0.034
5	4	1	4	3	2	9478.972	0.032
8	3	6	7	2	5	9485.668	-0.045
6	4	3	5	3	2	10143.887	0.077
6	4	2	5	3	3	10146.799	-0.001
7	4	4	6	3	3	10805.957	-0.007
7	4	3	6	3	4	10815.038	0.067
18	1	18	17	1	17	11143.217	-0.035
5	5	1	4	4	0	11233.073	0.033
8	4	5	7	3	4	11461.791	-0.018

8	4	4	7	3	5	11484.375	0.006
6	5	2	5	4	1	11900.776	-0.002
9	4	6	8	3	5	12107.219	0.026
9	4	5	8	3	6	12156.722	-0.039
15	3	13	14	2	12	12402.102	-0.098
7	5	3	6	4	2	12568.098	0.036
10	4	7	9	3	6	12736.467	-0.018
10	4	6	9	3	7	12834.995	-0.037
6	6	1	5	5	0	13655.049	-0.024
9	5	5	8	4	4	13899.334	-0.035
9	5	4	8	4	5	13900.748	-0.047

Table S3. Measured frequencies (values in MHz) for the rotational transitions of the *syn*/T/cc/t conformer of NAcGal

J'	K'_{-1}	K'_{+1}	J''	K''_{-1}	K''_{+1}	ν_{obs}	$\nu_{obs} - \nu_{calc}$
4	3	1	3	2	2	7119.725	0.065
5	3	2	4	2	3	7804.549	0.028
6	4	3	5	3	2	10224.033	-0.066
6	4	2	5	3	3	10226.912	0.057
7	4	4	6	3	3	10890.862	-0.024
7	4	3	6	3	4	10899.203	0.017
8	4	5	7	3	4	11551.746	-0.043
8	4	4	7	3	5	11572.511	-0.071
5	5	1	4	4	0	11324.534	0.073
6	5	2	5	4	1	11996.467	0.002
7	5	3	6	4	2	12667.948	-0.095
6	6	1	5	5	0	13766.392	0.056
12	0	12	11	1	11	7501.419	0.032
12	1	12	11	0	11	7648.948	0.011
11	2	9	10	1	10	13190.140	0.005
